# *ALK-R3HDM1* and *EML4-ALK* fusion as a mechanism of acquired resistance to gefitinib: A case report and literature review

**DOI:** 10.3389/fonc.2022.1010084

**Published:** 2022-10-31

**Authors:** Zhu Zeng, Tao Wang, Junjun He, Yuehong Wang

**Affiliations:** ^1^ Department of Respiratory Diseases, Thoracic Disease Center, The First Affiliated Hospital, Zhejiang University School of Medicine, Hangzhou, Zhejiang, China; ^2^ Department of R&D, Hangzhou Repugene Technology Co., Ltd., Hangzhou, China; ^3^ Zhejiang Provincial Key Laboratory of Pancreatic Disease, The First Affiliated Hospital, Zhejiang University School of Medicine, Hangzhou, China

**Keywords:** alectinib, *ALK* fusion, EGFR-TKI resistance, lung cancer, non-reciprocal/reciprocal *ALK*

## Abstract

We report a case with a novel *ALK-R3HDM1* and *EML4-ALK* dual fusion that might be a delicate mechanism for the acquired resistance of epidermal growth factor receptor (EGFR)–tyrosine kinase inhibitor (TKI). A patient with *EGFR* L858R lung adenocarcinoma developed disease progression after 72.7 months of gefitinib therapy; rebiopsy was done, and next-generation sequencing showed the disappearance of the previous *EGFR* mutations. In addition, two new *ALK* fusions emerged, indicating that the emergence of dual ALK rearrangement may be the underlying mechanism of gefitinib resistance. The patient exhibits an excellent response to second-line alectinib treatment with a significant clinical benefit and a high quality of life. Finally, we summarized previous studies in which *ALK* fusion is a required resistance mechanism to EGFR-TKI.

## Introduction

Epidermal growth factor receptor (*EGFR*) mutations and anaplastic lymphoma kinase (*ALK*) fusion represent two distinct subgroups of lung cancer, which are conventionally considered mutually exclusive (1, 2). Gefitinib has been approved as a first-line tyrosine kinase inhibitor (TKI) for treating EGFR-mutant patients. Despite the efficacy of gefitinib, disease relapse or progression is inevitable (3). The most common resistance mechanism is the secondary EGFR mutation of T790M in exon 20 (4). Recently, a few cases have been reported that required *ALK* rearrangement as a mechanism for EGFR-TKIs (5–7). Nevertheless, the newly emerged *ALK* fusion always coexists with the pre-existing *EGFR* mutation, and the authors concluded that the regimen of EGFR-TKI combined with ALK-TKI achieved a more satisfactory efficacy than monotherapy (5). Herein, we reported the first case of a novel *ALK-R3HDM1* and *EML4-ALK* double fusion as an acquired resistance mechanism to gefitinib with the disappearance of the original *EGFR* mutations and responding to alectinib. Consent for publication in print has been obtained from the patient.

## Case presentation

**Figure 1:**
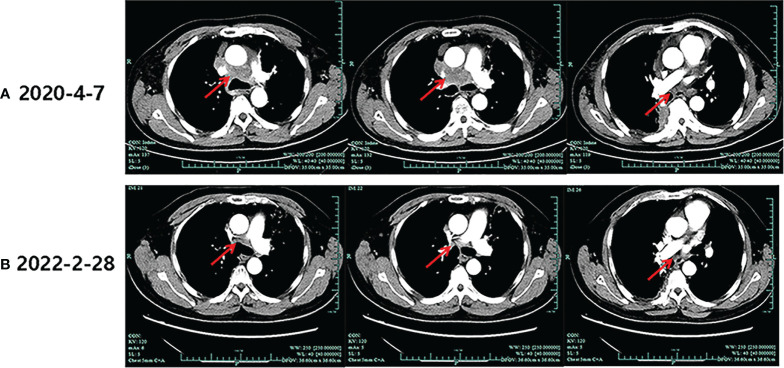
Imaging evolution of the patient. **(A)** The baseline computed tomography scan of alectinib treatment. **(B)** Computed tomography scan of the last follow-up of alectinib treatment.

**Figure 2:**
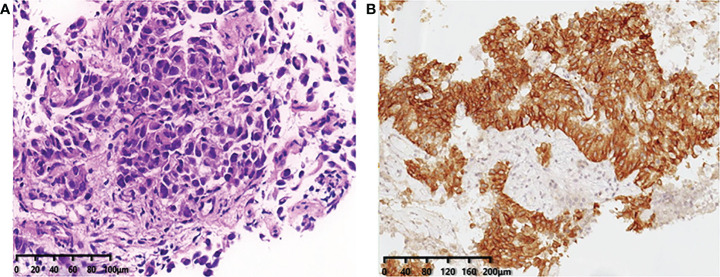
**(A)** Immunohistochemistry confirms the pathological typing of lung adenocarcinoma (×20). **(B)** Immunohistochemical staining of ALK expression (Ventana anti-ALK D5F3 clones, Roche-Ventana), and the pathologic assessment was positive (×20), localized in the cytoplasm. .

**Figure 3:**
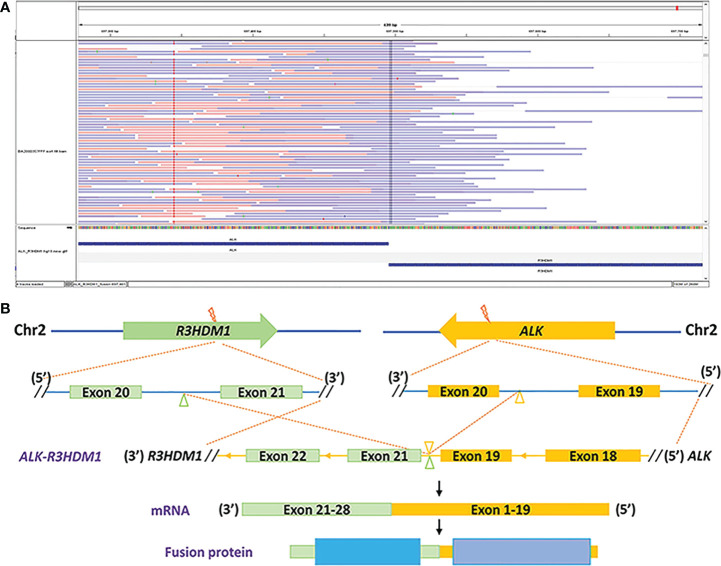
Identification of the *ALK-R3HDM1* fusion by next-generation sequencing. **(A)** Integrated Genomics Viewer snapshot demonstrating the *ALK-R3HDM1* translocation. **(B)** The schematic structure of the genomic DNA sequence shows the *ALK-R3HDM1* fusion points.

**Figure 4:**
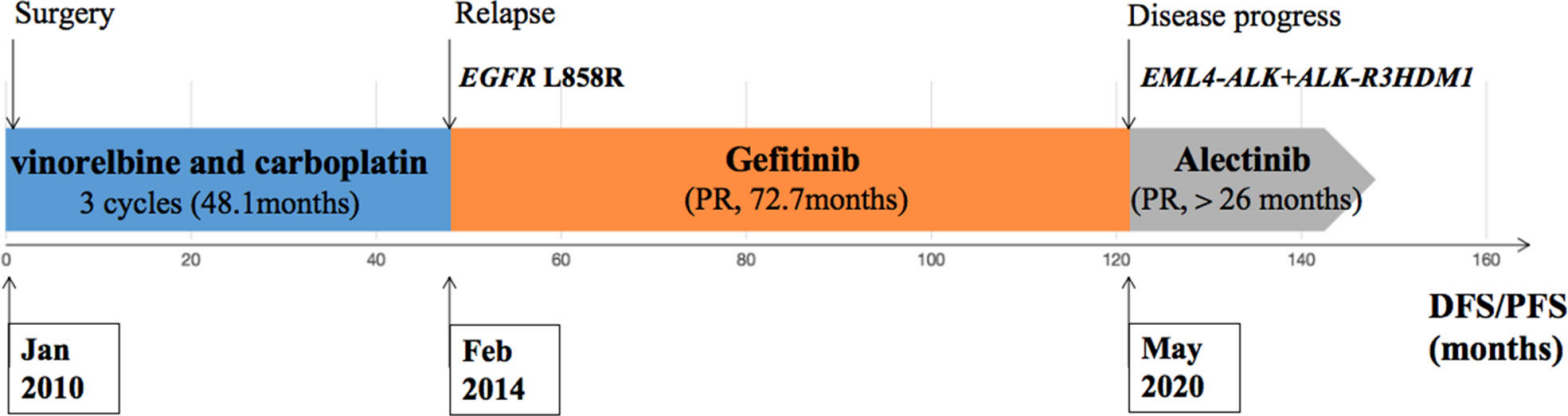
Description of the clinical course and pertinent molecular findings for the patient presented.

During his routine examination, a 65-year-old, never-smoker Chinese man was found with a nodule in the right lower lobe. The patient was assessed as acceptable for surgery, and he underwent radical resection on 28th January 2010. The final diagnosis was moderately differentiated lung adenocarcinoma (papillary mixed mucinous subtype) staged as pT1N1M0 IIA. Despite three cycles of the adjuvant chemotherapy of vinorelbine combined with carboplatin (he cannot tolerate the last cycle of chemotherapy), the patient developed multiple bilateral pulmonary in February 2014. Genetic analysis was performed in the tumor tissue of surgical specimens with *EGFR* exon 21 L858R mutation, *ALK/ROS1/KRAS/BRAF*-negative (real-time PCR for *EGFR/KRAS/BRAF* mutation testing, IHC for *ALK* fusion testing, and FISH for *ROS1* fusion testing). After first-line gefitinib treatment, tumors shrink and achieve partial response (PR). The patient suffered from increasing puffy face and shortness of breath after 72.7 months of treatment with gefitinib. CT scan showed multiple supraclavicular and intrapulmonary lymph node metastases (**Figure 1**). Meanwhile, an ultrasonographic (US) scan on superficial lymph nodes was performed for staging; the patient had supraclavicular lymphadenopathy and received a supraclavicular lymph node biopsy. Immunohistochemistry (IHC) indicated that this lymph node was metastatic lung adenocarcinoma with *ALK-*positive (**Figure 2**). Moreover, next-generation sequencing (NGS) approved that *ALK-R3HDM1*(A19: R21) and *EML4-ALK* (E6: A20, variant 3) rearrangement coexisted in specimens from the supraclavicular lymph node (**Figure 3**) without *EGFR* mutation. The novel *ALK-R3HDM1* rearrangement with an abundance of 27.48% and the *EML4-ALK* fusion was identified at an abundance of 20%. Unfortunately, the specimens acquired by bronchoscopy cannot meet the requirements for genetic testing, and we failed to validate the genetic profile in the lung. Thereafter, the patient received oral alectinib 600 mg bid as second-line therapy from 5 May 2020, with PR as the best response (**Figure 1**), and continued to receive clinical benefits from treatment. There is no clinical and radiological evidence of disease progression. The duration of response (DOR) of alectinib is over 26 months (**Figure 4**).

## Discussion and review of the literature

Numerous researchers have demonstrated that the acquired resistance mechanisms to EGFR-TKI were highly heterogeneous. The secondary *EGFR* mutations, alternative pathways activation and small-cell lung cancer transformation, are associated with resistance to EGFR-TKIs (8). Recent reports reveal the emergence of *de novo* receptor tyrosine kinase (RTK) fusions, including *ALK* rearrangement, as resistance mechanisms to EGFR-TKI (7). Herein, we presented a case with *ALK-R3HDM1* and *EML4-ALK* dual rearrangement set as a novel acquired resistance mechanism to gefitinib. To the best of our knowledge, this is the first study to report dual *ALK* rearrangements that participated in acquired resistance to gefitinib, with the original *EGFR* mutation disappearing in rebiopsy tumor tissue.

Previous studies clarified that *ALK* fusion is a resistance mechanism to EGFR-TKIs, but the prevalence of newly acquired *ALK* fusions developed during different EGFR-TKI treatments might be distinct; it could be found at a higher rate with osimertinib resistance and extremely low in first-generation EGFR-TKIs (6, 7). **Table 1** summarizes 12 cases that might indicate the novel mechanism of *ALK* rearrangement involvement in acquired resistance to EGFR-TKI. According to NGS testing after EGFR-TKI resistance, all these patients harbored an emerging *ALK* rearrangement that coexists with pre-existing *EGFR* mutations. Among these patients, 75% (9/12) of them have detected *ALK* fusion after the acquired resistance to osimertinib; only one patient (8.3%, 1/12) found that *ALK* rearrangement may serve as a molecular mechanism underlying acquired resistance to first-line TKI (erlotinib). It is unknown whether this potential enrichment of acquired fusions is related to the more potent *EGFR* inhibition of osimertinib or the later-line setting after multiple lines of EGFR inhibition.

**Table 1:** Details of published cases of novel ALK rearrangement attributed to acquired resistance to the epidermal growth factor receptor–tyrosine kinase inhibitor.

Case	Age/Sex	Genetic profiles before EGFR-TKI		Genetic profiles after EGFR-TKI				
		*EGFR* mutation	Treatment	*EGFR* mutation	*ALK* Fusion	OtherMutation	TreatmentAfter *ALK*Detection	PFS(months)
1#6	80/M	L861Q	Afatinib	L861Q, T790M	*EML4-ALK*	EGFR amp, KRAS G12D	Osimertinib	NA
2#6	55/F	T790M, L844V, L858R	Osimertinib	L844V, L858R	*EML4-ALK*	EGFR amp	NA	NA
3#6	57/F	E746-A750del, T790M	Osimertinib	E746-A750del,T790M,C797S,	*EML4-ALK*	EGFR amp	NA	NA
4#6	64/F	L858R, T790M	Afatinib resistance, then switch to osimertinib	L858R	*STRN-ALK*	NA	Osimertinib	NA
5#6	59/M	E746-A750del	Erlotinib	E746-A750del, T790M	*CEBPZ-ALK*	NA	Crizotinib	NA
6#13	66/M	19 del followed by acquired T790M	Gefitinib followed by osimertinib	19 del, T790M, C797G	*EML4-ALK*	PI3KCA	Crizotinib	1
7#14	42/M	19 del followed by acquired T790M	Gefitinib followed by osimertinib	19 del	*STRN-ALK*	None	Gefitinib + crizotinib	6
8#5	60/F	19 del	Gefitinib resistance, then switch to osimertinib	19 del	*EML4-ALK*	CDK4 Amp	Osimertinib + crizotinib	6
9#15	70/F	L858R followed by acquired T790M	Erlotinib followed by osimertinib	L858R, T790M	*PLEKHA7-ALK*	MDM2 Amp	Osimertinib + alectinib	6
10#7	65/F	19 del followed by acquired T790M	Erlotinib followed by osimertinib + necitumumab	19del, T790M	*EML4-ALK*	None	Osimertinib + alectinib	NA
11#7	68/F	L858R followed by acquired T790M	Erlotinib followed by osimertinib	T790M	*EML4-ALKJ*	None	Alectinib	NA
12#16	46/F	19 del(second and third line no molecular detection)	Erlotinib(second and third line received chemotherapy)	19 del, L747S	*EML4-ALK*	None	Osimertib + crizotinib	NA

EML4-ALK, echinoderm microtubule–associated protein like 4-anaplastic lymphoma kinase; EGFR, epidermal growth factor receptor; del, deletion; TKI, tyrosine kinase inhibitor; PFS, progression-free survival; M, male; F, female; KRAS, Kirsten rat sarcoma viral oncogene homolog; amp, amplication; NA, not available, PI3KCA, phosphatidylinositol-4,5-bisphosphate 3-kinase catalytic subunit alpha.

It has been previously reported that approximately 1.3%–1.6% of non-small cell lung cancer (NSCLC) patients harbor concomitant *EGFR* mutations and *ALK* rearrangements in the baseline (9, 10). Hence, it is critical to identify truly acquired alterations from concomitant genetic abnormalities in pretreatment tissue. In this case, the initial tumor tissue from the primary surgical specimens showed only *EGFR* L858R mutation and negative *ALK* fusion, while rebiopsy from the metastatic supraclavicular lymph node at the progression of gefitinib treatment revealed the newly emerged fusions in *ALK* without the original *EGFR* mutation, indicating that the tumor progression was most likely due to the emergence of concurrent *ALK- R3HDM1* and *EML4-ALK* fusions. Although the new *ALK* alteration emerged in previous studies as a resistance mechanism to EGFR-TKI, the original driver gene *EGFR* mutation was preserved (**Table 1**). Hence, previous studies indicated that dual TKI treatment might benefit EGFR-TKI-acquired resistant NSCLC patients induced by ALK rearrangement rather than the ALK inhibitor alone (5). In the current case, the absence of *EGFR* gene mutation in the rebiopsy tissue suggested that *ALK* drives oncogenesis after EGFR-TKI resistance. Hence, the patient received an exceptional response to treatment with alectinib monotherapy. Unfortunately, we only performed the NGS analysis of supraclavicular lymph nodes and could not present the detailed genetic profile by NGS in the primary lung tumor since the surgical specimens are not available.

Recently, numerous *ALK* fusion partners have been identified in NSCLC patients, and the response is significantly varied by the fusion partner (11). Herein, we first report a novel fusion partner *RH3D1*, in an NSCLC patient with *EML4-ALK* simultaneously. Previous studies defined these fusions as non-reciprocal/reciprocal *ALK*, harboring concurrent *ALK* fusions with at least one 3′-*ALK* fusion and one 5′-*ALK*, which may not affect inhibitor response (12). However, Zhang et al. reported that non-reciprocal/reciprocal *ALK* fusion is a negative prognostic factor for NSCLC patients harboring *ALK* fusion and a negative predictive factor for crizotinib treatment (12). This is the first case to assess the response of ALK-TKI in acquired non-reciprocal/reciprocal *ALK* fusion to EGFR-TKI. According to a previous study, the median PFS of patients with non-reciprocal/reciprocal ALK fusion who received first-line crizotinib was 6.1 months(n = 23, 95%CI: 5.0–11.0 months). However, our patient exhibits an excellent response to second-line alectinib treatment; the duration of response (DOR) is more than 26 months with a significant clinical benefit and a high quality of life. There are two possible reasons for this: firstly, all dual fusions reported by Yang and his colleagues were primary driver genes, whereas fusions in our patients were required after EGFR-TKI resistance, which may be attributed to a more complex molecular mechanism after drug resistance; secondly, alectinib may be more effective in the non-reciprocal/reciprocal ALK fusion than crizotinib.

It would be interesting to know how these dual *ALK* rearrangements contribute to the excellent response of alectinib in this patient. Further mechanistic studies are warranted to elucidate their intracellular function. Our report is also limited by the lack of tumor tissue to differentiate second primary lung cancer from intrapulmonary metastases lung cancer. A more comprehensive analysis of the primary and metastatic lesions in the genome may provide more clues to the above questions. However, owing to the lack of tumor specimens for further analyses, some crucial information was not available.

## Conclusion

In summary, we report that an NSCLC patient who experienced gefitinib resistance and was shown to harbor that the emerging non-reciprocal/reciprocal *ALK* rearrangements achieved satisfactory efficacy with alectinib monotherapy. The finding of this new *ALK-R3HDM1* fusion may provide a more molecular profiling of patients with non-reciprocal/reciprocal *ALK* fusions to optimize the therapeutic strategy. Furthermore, the regimen of alectinib may achieve a more satisfactory efficacy than crizotinib in patients with non-reciprocal/reciprocal *ALK* fusions.

## Data availability statement

The original contributions presented in the study are included in the article/supplementary material. Further inquiries can be directed to the corresponding author.

## Ethics statement

This study was approved by the Ethics Committee of the First Affiliated Hospital, Zhejiang University School of Medicine. The patients/participants provided their written informed consent to participate in this study.

## Author contributions

YW designed the case report, ZZ drafted the manuscript, TW and JH are responsible for data analysisand graphing. All authors contributed to the article and approved the submitted version.

## Funding

This research was supported by National Natural Science Foundation of China under Grant Number 82002411 and the key projects of Natural Science Foundation of Zhejiang Province under Grant Number LZ22H160011.

## Acknowledgments

The authors would like to thank all study participants and their families and are grateful for the collaboration of The First Affiliated Hospital, College of Medicine, Zhejiang University, and their staff.

## Conflict of interest

Author TW was employed by Hangzhou Repugene Technology Co., Ltd.

The remaining authors declare that the research was conducted in the absence of any commercial or financial relationships that could be construed as a potential conflict of interest.

## Publisher’s note

All claims expressed in this article are solely those of the authors and do not necessarily represent those of their affiliated organizations, or those of the publisher, the editors and the reviewers. Any product that may be evaluated in this article, or claim that may be made by its manufacturer, is not guaranteed or endorsed by the publisher.
